# Mutations of Recombinant Aquaporin-4 Antibody in the Fc Domain Can Impair Complement-Dependent Cellular Cytotoxicity and Transplacental Transport

**DOI:** 10.3389/fimmu.2018.01599

**Published:** 2018-07-13

**Authors:** Simone Mader, Lior Brimberg, John N. Soltys, Jeffrey L. Bennett, Betty Diamond

**Affiliations:** ^1^The Feinstein Institute for Medical Research, The Center for Autoimmune, Musculoskeletal and Hematopoietic Diseases, Northwell Health System, Manhasset, NY, United States; ^2^Medical Scientist Training and Neuroscience Graduate Training Programs, University of Colorado Denver School of Medicine, Aurora, IL, United States; ^3^Department of Neurology, Program in Neuroscience, University of Colorado Denver School of Medicine, Aurora, IL, United States; ^4^Department of Ophthalmology, Program in Neuroscience, University of Colorado Denver School of Medicine, Aurora, IL, United States

**Keywords:** maternal antibody, transplacental transport, FcRn, aquaporin-4 IgG, complement-dependent cytotoxicity

## Abstract

Maternal antibodies provide protection for the developing fetus. Transplacental transport of pathogenic autoantibodies might pose a risk for the developing fetus. The transport of antibodies across the placenta to the fetal circulation occurs through the neonatal Fc salvage receptor (FcRn). During gestation, maternal autoantibodies are able to penetrate the embryonic brain before a functional intact blood–brain barrier is established. Brain-reactive antibodies to the water channel protein aquaporin-4 (AQP4) are a hallmark finding in neuromyelitis optica (NMO), a neurological disease that predominantly affects women, many of whom are of childbearing age. AQP4–IgG mediate astrocytic injury in a complement-dependent fashion. Recent studies suggest these antibodies contribute to impaired pregnancy outcome. The aim of the study was to investigate the transplacental transport as well as FcRn binding of a monoclonal AQP4–IgG cloned from an NMO patient (wild-type antibody) compared to five different mutated Fc domain of this antibody containing single amino acid substitutions in the Fc region. All of the Fc-mutated antibodies lack complement-dependent cytotoxicity. Four of the five Fc-mutated antibodies showed limited transplacental transport *in vivo*. Three mutated Fc with impaired transplacental transport showed persistent binding to rodent FcRn at pH 6 but also at pH 7.2, suggesting that limited transplacental transport could be due to diminished release from FcRn. One mutated Fc with modestly limited transplacental transport showed diminished binding to FcRn at pH 6. This study suggests that mutated Fc with intact transplacental transport may be used to study antibody effector functions and Fc with limited transport may be used as a carrier to deliver therapies to pregnant woman, while sparing the developing fetus.

## Introduction

Antibodies play an essential role in health. During development, they have a crucial role in providing the fetus with passive protection against pathogens. The transfer of immunity from the mother to the fetus is dominated by IgG transport across the placenta (1). Maternal antibodies cross the placenta beginning around the second trimester of the pregnancy (2). Under routine conditions, the neonatal Fc salvage receptor (FcRn) regulates antibody half-life; during pregnancy it also mediates transcytosis of IgG from the maternal to the fetal circulation (3). Following internalization of IgG by pinocytosis, the binding of IgG antibodies to FcRn occurs in acidic early endosomes at pH 6 (4, 5). While unbound IgG is degraded, FcRn–IgG complexes are routed away from the lysosomal pathway and intact IgG is released at physiological pH 7.2 when phagolysosome fuses with the plasma membrane (6–9). Therefore, both IgG binding to FcRn as well as its release from FcRn can influence transplacental transport of antibodies.

Maternal IgG has a protective role during pregnancy, yet pathogenic autoantibodies are also transported to the fetus and may impair fetal development, resulting in either transient or permanent damage to the developing fetus. The severity of the insult is determined by several factors, such as the antigenic specificity of the autoantibodies, maternal IgG concentration, and the gestational stage at which the antigen is expressed.

It is well appreciated that a number of neonatal disorders are associated with *in utero* exposure to pathogenic autoantibodies. For example, transport of anti-acetylcholine receptor antibodies from mothers with myasthenia gravis can result in either transient myasthenia gravis or in permanent defects in the neonate (10–12) and transplacental transport of anti-Ro antibodies in mothers with autoimmune disorders can result in fetal heart block or neonatal lupus (13).

Pathogenic autoantibodies are not solely present in individuals with autoimmune diseases; they are also present in a subgroup of healthy individuals. In fact, mothers of children with autism spectrum disorder have an increased frequency of brain-reactive antibodies (14), and *in utero* exposure to brain-reactive antibody such as antibody to the neuronal voltage-gated potassium channel Caspr2, results in neurodevelopmental impairment and permanent behavioral alterations in mice (15). Since the fetal blood–brain barrier (BBB) is permeable to maternal IgG in mice until gestational day E16.5 (16), and in humans most likely until the third trimester, the developing brain is a potential target for maternal autoantibodies, even when the mother experiences no clinical symptoms.

Studies of the pathogenicity of maternal brain-reactive antibodies often employ a model in which the antibodies are injected into a pregnant animal at a gestational stage when the antigen of interest is expressed and the embryonic BBB allows penetration of the antibody to the fetal brain (15, 17–19). Antibodies with Fc mutations that alter effector function need to be analyzed for transplacental transport in order to determine whether they can be used in studies to address the mechanism of action of maternal antibodies.

Here, we describe a brain-reactive antibody directed to AQP4 (AQP4–IgG). Neuromyelitis optica (NMO) is an inflammatory, demyelinating neurological disease that predominantly affects the optic nerve, spinal cord, and brain. AQP4–IgG testing is included as a diagnostic marker of the disease (20) and several studies in rodent models have demonstrated its pathogenicity (21, 22). AQP4–IgG is thought to result in tissue damage primarily through complement-dependent cytotoxicity (CDC), but other effector mechanisms such as antibody dependent cell-mediated cytotoxicity may also appear to play a role in the disease (23, 24).

Recently, several case reports have suggested that AQP4–IgG can have a negative impact on pregnancy outcomes (25); further studies are needed to address this question. It is reported that women with NMO and AQP4–IgG have a higher rate of miscarriages and suffer more often from preeclampsia (25). Whether placental or fetal damage results from anti-AQP4 IgG exposure and whether the mechanism of injury is complement dependent is not known.

In this paper, we compared transplacental transport of a human monoclonal AQP4–IgG1 antibody cloned from a patient with NMO (22), and five variants containing different point mutations in the Fc domain designed to reduce complement activation. All the antibodies with mutated Fc were deficient in CDC *in vitro*; however, they differed in their ability to cross the placenta. At the time point when less transplacental transport was observed, all of the antibodies were present in the blood at similar concentrations. Yet, one mutated Fc with impaired transplacental transport showed diminished binding to mouse FcRn at pH 6, while the other three mutated Fc antibodies with severely impaired transplacental transport showed enhanced binding to FcRn at pH 7.2, suggesting that the antibodies are not as efficiently released from FcRn.

This study emphasizes that single point mutations in the Fc region can affect transplacental transport. Our findings are important for the study of effector mechanisms by which maternal antibodies mediates fetal pathology. In addition, Fc variants with altered transport might also provide clinical application.

## Materials and Methods

### Human Monoclonal Antibodies

Human monoclonal AQP4–IgG (rAb-53) was cloned from a plasmablast recovered from the cerebrospinal fluid of an NMO patient, produced, and purified as previously described (22). Isotype control (Ctl) was a non-brain binding antibody directed against measles virus (2B4-IgG) (22). Recombinant human AQP4–IgG (rAb-53) with mutated Fc sequences was generated by site directed mutagenesis. Mutation nomenclature is according to the EU index as described in Kabat et al. (26) K322A (KA) (27, 28), P329A (PA) (28), N297D (ND) (29), D270A (DA) (28), and P331G (PG) (28).

### Cell-Based Assay (Immunofluorescence)

To confirm binding of wild-type and Fc-mutated antibodies to AQP4, we performed a live cell-based assay as previously described (30) with some modifications. Briefly, human embryonic kidney cells, HEK293T/17 (ATCC), were cultured on poly-d lysine (Sigma) coated glass cover slips (TED PELLA, Inc.) in 24-well plate. 24 h after seeding the cells, HEK293T/17 cells were transiently transfected using FuGene (ProMega) with AQP4 (M23 isoform) fused at its C terminus to an emerald green fluorescent protein. Two days after transfection the assay was performed.

Antibodies (Ctl-IgG, AQP4-IgG, DA-IgG, KA-IgG, ND-IgG, PA-IgG, and PG-IgG), 5 µg/ml, were diluted in HBSS/10% FBS and incubated with cells for 1 hour at 4°C. Cells were washed three times with HBSS/10% FBS and incubated with a secondary anti-human antibody Alexa 594 (Invitrogen) for 30 min at room temperature. Cells were washed three times, fixed with 4% paraformaldehyde (PFA) for 15 min, washed again with HBSS and coverslips were mounted on a slide. Pictures were acquired at 20× magnification using Axio-Imager Z-1 software (Axio-Vision, Zeiss, Oberkochen, Germany). Untransfected cells served as control. DAPI positive cells, i.e., dead cells, were excluded from analysis.

### Infrared Labeling of Human Monoclonal Antibodies

All human monoclonal antibodies were labeled with the IRDye 800 CW protein labeling kit following the manufacturer’s instructions (Microscale, Invitrogen). Briefly, the pH level was adjusted to 8.5 by adding 1 M potassium phosphate buffer to 200 µl of antibody solution (1 mg/ml of IgG in PBS). 1.4 µl of dissolved dye was added to the antibody solution and the mixture was incubated for 2 h at room temperature. Subsequently, free dye was removed using Zeba Spin Desalting columns according to the manufacturer’s instructions. Centrifuge columns were washed in PBS three times, and excess wash solution was removed by dry spin at 1,500 *g*. The columns were placed in a new collection tube and 100 µl of the sample was applied to each column. The tube was centrifuged at 1,500 *g* for 2 min to collect the sample. Integrity of labeled antibodies was confirmed using a non-reducing 12% Bis-Tris Protein Gel (NuPage, Invitrogen) according to manufacturer’s instruction. Gel was stained with Coomassie Brilliant Blue Staining (Invitrogen).

To confirm intensity of labeling, dot blots were performed with serial dilutions of infrared labeled antibodies, on a nitrocellulose membrane (Biorad, 0.45 µm), drying the membrane and scanning for signal intensity. Starting dilution was 20 µg/ml of infrared labeled antibody. Membrane analysis was performed using the Odysey infrared imaging system (LICOR).

### Cell-Based Assay (for Infrared Labeled Antibodies)

Binding to HEK 293 cells expressing AQP4 was confirmed by cell-based assay as follows using HEK 293T/17 cells expressing AQP4 seeded in a 96-well tissue culture plate as previously described (30). In short, cells were incubated with the infrared labeled antibodies (1 µg/ml) in triplicates and incubated for 1 h at 4 C°. Following three washing steps with PBS/10% FBS, the liquid was removed and the plate was immediately scanned using an infrared imaging system (Odyssey-Licor). The signal intensity of transfected and untransfected cells was compared for all antibodies. The infrared labeled monoclonal antibody Ctl-IgG which does not bind AQP4, was used as control for background binding. Untransfected cells were used as controls.

### Study Approval

All animal experiments were performed in accordance with the National Institutes of Health Guidelines under protocols reviewed and approved by the Institutional Animal Care and Use Committee of the Feinstein Institute for Medical Research, Northwell Health, Manhasset, NY, USA.

### Mice and Injection of Infrared Labeled Antibody

C57BL/6 mice (6–8 weeks old) were obtained from the Jackson Laboratory. For timed pregnancy, 2 female mice and 1 male mouse were housed together for 14 h. The time when the male mouse was removed from the cage was designated embryonic (E) day 0.5. Infrared labeled IgG (60 µg) was administered by retro-orbital injection in 200 µl volume (300 µg/ml) to time-pregnant mice under light anesthesia at E14.5. Embryos were harvested at E15.5 as described below. The degree of transplacental transport was at least confirmed for 2 litters to each antibody.

### Infrared Scanning of Embryos

24 h after injection of the infrared labeled antibodies (Ctl-IgG, AQP4-IgG, DA-IgG, KA-IgG, ND-IgG, PA-IgG, and PG-IgG) we harvested the embryos with placenta attached and scanned the embryo and placenta. An embryo not exposed to an antibody was included as an additional control to confirm absence of an infrared signal. Scan setting was kept constant for all litters. Infrared signal intensity was quantified for each embryo using the Odyssey Infrared Imaging System (LI-COR, Linconln, NE, USA) and data are presented as ratio of pixel intensity of each embryo normalized to the average pixel intensity of an AQP4 litter run on the same scan to account for variability within scans. Embryos were derived from 2 litters for each antibody.

### Antibody Half-Life in Serum of Mice

10–20 µg of human IgG (Ctl-IgG, AQP4-IgG, KA-IgG, ND-IgG, PA-IgG, and PG-IgG, noninfrared labeled) was injected retroribitally to non-pregnant female C57BL/6 mice (6–8 weeks old). To determine levels of human IgG in the serum, mice were euthanized at indicated time points, and blood was collected through the orbital sinus. Blood was spun down at 10,000 *g* for 15 min and serum was removed and frozen at −20°C until use. Three animals were analyzed for each antibody and blood was drawn at different time points (time 0; 3 min after injection, time 0.5, 1, 2, 4, 6, 24, 48, and 72 h). Serum concentration of human IgG is presented (μg/ml). Human IgG levels were measured by ELISA as described below and presented as percentage of human IgG at 24 h relative to time 0.

### ELISA for Measuring IgG Concentrations

We used an ELISA to measure human IgG concentrations prior to injections and at different time points after injection in the serum of mice.

ELISA plates (Costar) were coated with goat anti-human IgG using a concentration of 10 µg/ml and incubated for 1 h at 37°C. The plates were washed two times with PBS/Tween and subsequently blocked with PBS/3% FBS for an hour at 37°C. Following two washing steps with PBS/Tween, samples were diluted in PBS/0.3% FBS and incubated at 37°C for 90 min. Human corresponding IgG (Ctl-IgG, AQP4-IgG, DA-IgG, KA-IgG, ND-IgG, PA-IgG, and PG-IgG) diluted to 200, 100, 50, 25, 12.5, 6.25, 3.125, and 1.56 ng/ml was added as a standard. Following four washing steps with PBS/Tween AP-conjugated secondary goat anti-human IgG antibody (1:1,000 dilution) was added for 1 h at 37°C. Plates were washed four times with PBS/Tween. Phosphatase developing solution was added and plates were read at 405 nm absorbance every 10 min. Concentration of each antibody prior to injection was also confirmed using nanodrop.

### Complement-Dependent Cytotoxicity

Complement-dependent cytotoxicity assay was performed as previously described (24). CHO cells stably expressing M23–AQP4 were plated at 37,500 cells/well and cultured overnight in CHO cell medium (10% FBS + 1% penicillin/streptomycin/amphotericin B in F12 media; Gibco). Cells were washed twice with pre-warmed F12 medium and incubated at 37°C for 60 min with medium alone, 5% pooled human serum (Complement Technology, Inc.), or Ab ± 5% pooled human serum. All Ab treatments were at 5 µg/ml and were chosen based on a titration curve previously performed (24). Cells were then washed in medium and treated for 15 min with 1 µM Calcein AM (CaAM; Invitrogen) to label live cells green and 1 µM propidium iodide (PI; Invitrogen) to label dead cells red. Images were taken on a Leica inverted fluorescence microscope. Cells were counted using ImageJ and the percentage of cell lysis was calculated as [PI positive cells/(PI positive + CaAM positive cells)] × 100%. Statistical analysis was performed using analysis of variance (ANOVA) and Dunnett’s test for multiple comparisons in GraphPad Prism 5 software.

### FcRn Binding Using Octet Fortebio

FcRn binding experiments were performed on an Octet K2 (PALL/ForteBio) instrument at 30°C, according to ForteBio technical note 19 and 34 (https://www.fortebio.com/literature.html). To measure FcRn binding, human monoclonal antibodies that were not infrared labeled were used and each antibody was analyzed using three independent times at two different pH levels, showing comparable results. Anti-human Fab-CH1 biosensors (PALL ForteBio) were loaded with monoclonal antibodies (6 µg/ml) in kinetic buffer (PALL ForteBio). The concentration of antibody was selected by an optimization step as described in technical note 19.

For association phase, mouse FcRn-Beta-2 microglobulin heterodimer protein (ImmuniTrack) was diluted with assay association and disassociation buffer (100 mM sodium phosphate, 150 mM Nacl, 0.05% Tween-20, pH 6) to concentrations ranging from 2,000 to 0 nM and transferred to solid-black 96-well plate (Greiner Bio-One). FcRn was allowed to bind to IgG loaded biosensors for 60 s. The dissociation phase was recorded in assay buffer pH 6.0 for 60 s.

The experiments at neutral pH were performed as described above except that PBS pH 7.2 was used for FcRn dilution, association, and dissociation.

All data were referenced with individual AQP4 wild-type or mutated Fc antibodies loaded biosensors incubated in assay buffer instead of FcRn, and with the biosensor loaded with only dilutant and various concentrations of FcRn. The sensorgrams were plotted and evaluated using data analysis software version 9.0 (PALL/ForteBio). Rmax, the maximum response determined from the fit of the binding data and Req, the calculated response at equilibrium that is determined from a fit of the binding data are presented. In addition, KD (M) and KD error are presented.

### Statistical Analysis

We performed ANOVA followed by Dunnett’s multiple comparisons test as well as Student’s *t*-test for datasets that were normally distributed. Otherwise, we performed Mann–Whitney test. All tests were performed with the Graph pad prism statistical software package. Values were considered significant for *p* < 0.05. Data are presented as mean and error bars represent standard error. All tests were two tailed.

## Results

### Binding to AQP4

**Figure 1:**
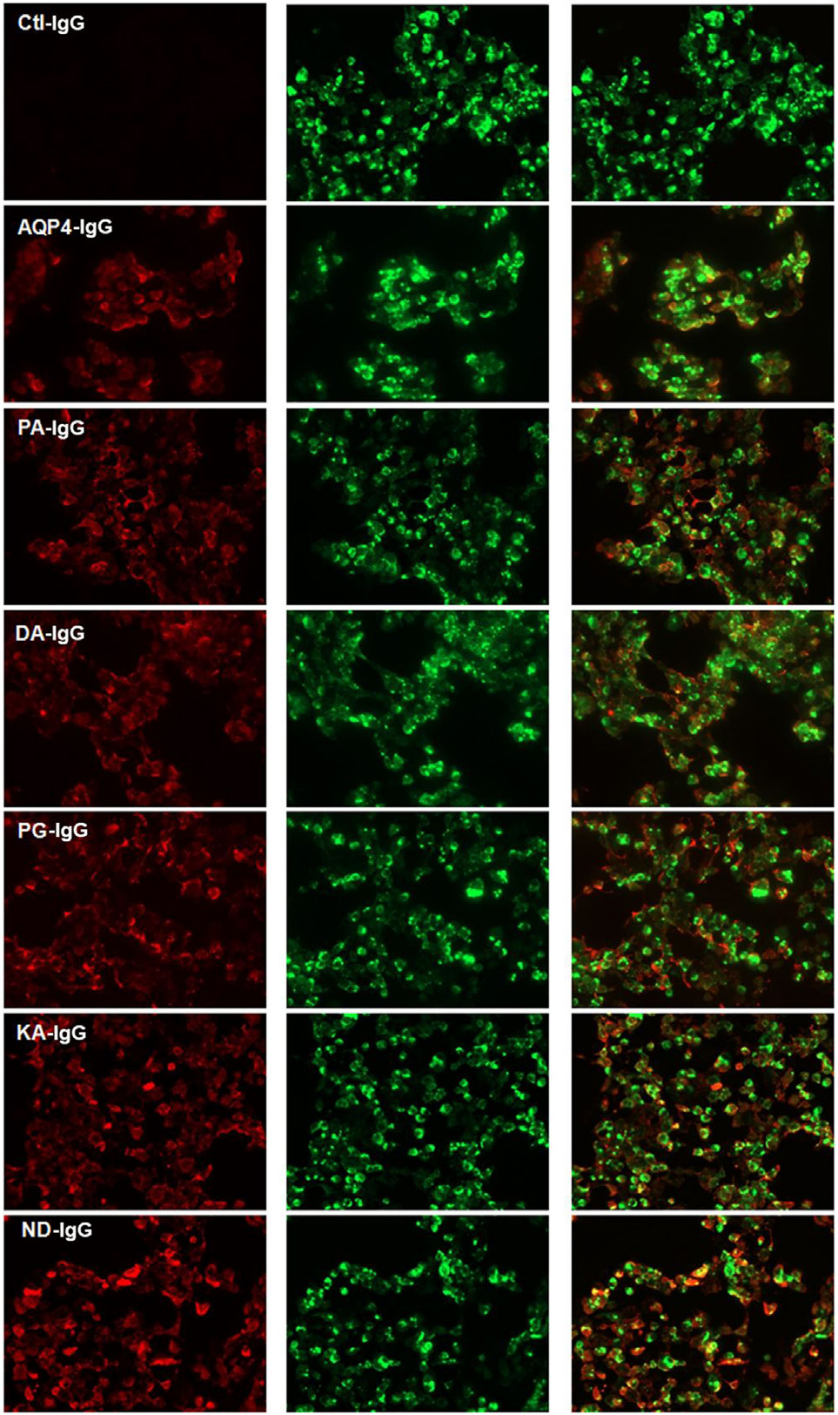
Cell-based assay demonstrating binding of AQP4–IgG and Fc-mutated AQP4–IgG to AQP4 transfected cells. Binding of infrared labeled AQP4–IgG and all Fc-mutated antibodies DA-IgG, KA-IgG, PA-IgG, and PG-IgG (shown in red) to HEK cells which express AQP4 fused with a green fluorescent protein (shown in green). The mutations do not interfere with the ability of the antibodies to bind AQP4. The isotype Ctl-IgG, does not bind to AQP4 transfected cells. None of the antibodies bind to non transfected cells (not shown).

Five Fc-mutated antibodies of an IgG1 anti-AQP4 antibody were generated, all with an identical light chain and heavy chain variable region, but each with a single amino acid substitution in the Fc region of the heavy chain. All Fc-mutated antibodies were tested for AQP4 binding. As seen in Figure 1, all mutated AQP4 antibodies bound to AQP4 in a live cell assay.

### Complement Activation

**Figure 2:**
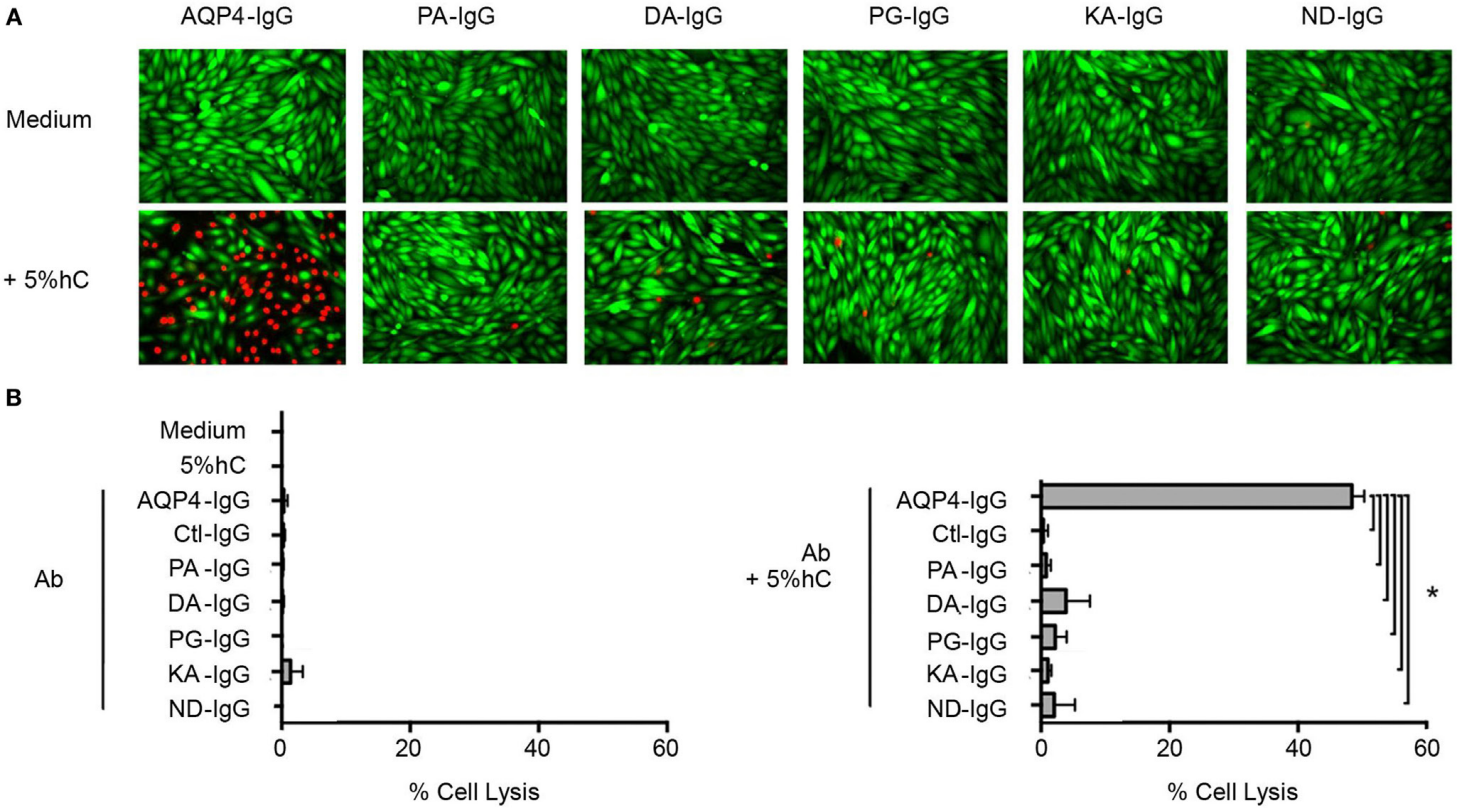
Antibody Fc domain mutations limit complement-dependent cytotoxicity activation by AQP4–IgG. **(A)** CHO cells expressing M23–AQP4 were incubated with 5 µg/ml monoclonal antibody in the absence (left row) or presence (right row) of 5% pooled human serum (hC) for 60 min at 37°C. Live cells are labeled with calcein AM, which is a cell viability dye (green); dead cells are labeled with propidium iodide (red). **(B)** Cell lysis (mean percentage ± SD) is plotted for medium alone, hC alone, and each monoclonal antibody without (top) or with (bottom) hC. Ctl-IgG is specific for measles virus nucleocapsid protein and serves as a negative control. *p < 0.0001; analysis of variance with Dunnett’s multiple comparisons test.

The mutations in the Fc region were designed to decrease CDC. We determined that each Fc-mutated antibody displayed altered complement activation compared to wild-type AQP4–IgG using an *in vitro* assay. The wild-type AQP4–IgG antibody activated complement as previously described (27). Each of the five Fc-mutated antibodies lacked the ability to activate the complement cascade, and exhibited diminished CDC (Figures 2A,B).

No cell lysis was observed with wild-type AQP4-IgG in the absence of human complement, or with isotype-matched Ctl-IgG antibody or medium containing human complement alone (Figure 2B; Table 1).

**Table 1:** AQP4–IgG-mutated antibodies with limited complement-mediated cytotoxicity.

Treatment	% Lysis (mean ± SD)
Medium	0.00 ± 0.00
5% hC	0.00 ± 0.00
Ctl-IgG	0.24 ± 0.14
Ctl-IgG + 5% hC	0.36 ± 0.36
AQP4-IgG	0.31 ± 0.31
AQP4-IgG + 5% hC	48.39 ± 0.97
PA-IgG	0.11 ± 0.11
PA-IgG + 5% hC	0.82 ± 0.34
DA-IgG	0.11 ± 0.11
DA-IgG + 5% hC	3.89 ± 1.87
PG-IgG	0.00 ± 0.00
PG-IgG + 5% hC	2.23 ± 0.87
KA-IgG	1.38 ± 0.86
KA-IgG + 5% hC	1.07 ± 0.28
ND-IgG	0.00 ± 0.00
ND-IgG + 5% hC	2.05 ± 1.62

Cell lysis of CHO cells expressing AQP4 (mean percentage ± SD) is presented for medium alone, human complement (hC) alone, and Ab without (top) or with (bottom) hC. Ctl-IgG antibody is specific for measles virus nucleocapsid protein and serves as a negative control. *p < 0.001, analysis of variance with Dunnett’s multiple comparison test.

### Transplacental Transport of Antibodies

**Figure 3:**
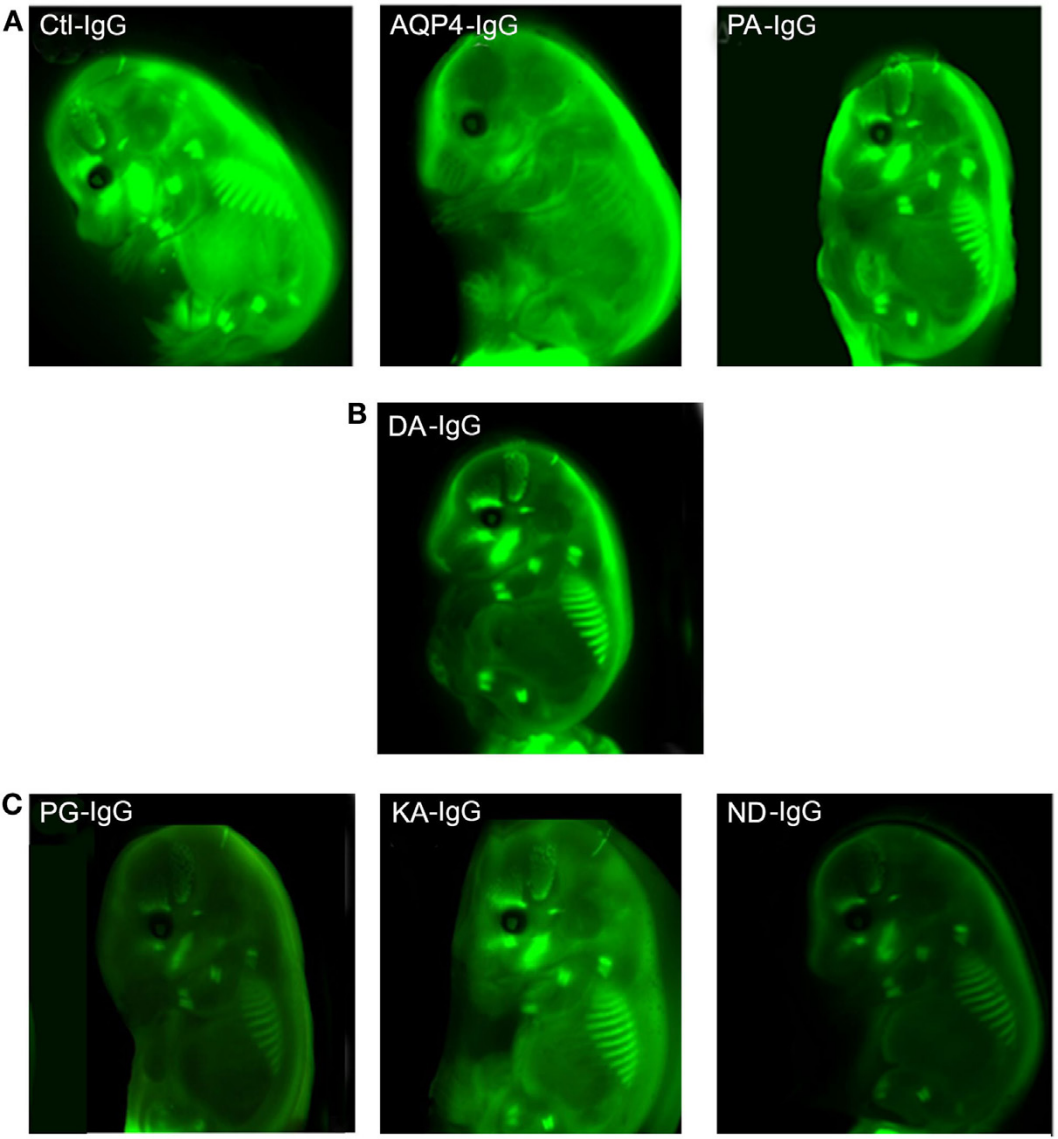
Effect of mutations of AQP4–IgG on transport across the placenta. Infrared labeled AQP4–IgG and its isotype control antibody (Ctl-IgG) as well as the Fc-mutated PA-IgG show an equal degree of transplacental transport in the embryo **(A)**. DA-IgG shows modestly diminished transport **(B)**. KA-IgG, ND-IgG, and PG-IgG have severely impaired transplacental transport **(C)**. Each antibody was tested in 2 litters at a concentration of 60 µg.

**Figure 4:**
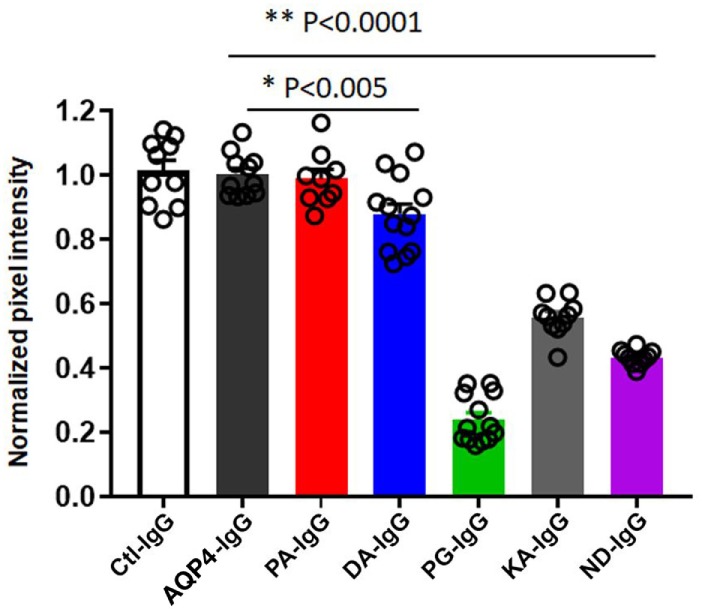
Quantification of transplacental transport of antibodies. Quanitification of optical intensity of the infrared signal (pixel intensity) from embryos exposed to maternal infrared labeled antibodies *in utero*. The values of each embryo is averaged and shown normalized to the average pixel intensity of an AQP4–IgG exposed litter present on the same scan to account for variability within scans (normalized pixel intensity). Transplacental transport of each antibody is shown for both litters. One-Way analysis of variance followed by Dunnett’s multiple comparisons test revealed that mice injected with PG-IgG, KA-IgG, and ND-IgG showed markedly reduced normalized infrared pixel intensity compared to AQP4–IgG (**p < 0.0001). DA-IgG showed a modest, but significant reduction, infrared pixel intensity compared to AQP4–IgG (*p < 0.005). Ctl-IgG, AQP4-IgG, and PA-IgG are not different (*p* > 0.8).

In order to investigate transplacental transport of AQP4–IgG and the five CDC Fc-mutated antibodies, we injected infrared labeled antibody intravenously into pregnant mice. Antibody was administrated by retro-orbital injection into pregnant dams on embryonic day E14.5 and fetuses were scanned for the presence of the infrared labeled antibody 24 h later, a time point when the embryonic BBB still allows antibody transport to the brain (16) (Figures 3 and 4; Figure S1 in Supplementary Material). Embryos derived from two litters from each antibody were compared to the transport of wild-type AQP4–IgG (Figure 4; Figure S1 in Supplementary Material). Whereas Ctl-IgG antibody, wild-type AQP4-IgG antibody and the Fc-mutated antibody PA-IgG showed equal transport to the fetus, four Fc-mutated antibodies exhibited reduced fetal transport; the impact ranged from modest (DA-IgG) to severe (KA-IgG, ND-IgG, and PG-IgG) (Figures 3 and 4; Figure S1 in Supplementary Material). The antibodies that entered the fetal circulation could all be observed in the fetal brain. Placentas showed similar levels of high infrared signal even for mutated antibodies with less transplacental transport, as shown for Fc-mutated ND-IgG in Figure S2 in Supplementary Material.

Reduced transplacental transport could not be explained by a difference in labeling of the antibodies or differences in binding to AQP4, since we confirmed that all antibodies had the same degree of labeling prior to injection (Figures S3A,B in Supplementary Material), and we confirmed that labeling had no effect on their ability to bind to AQP4 expressed by transfected HEK293T cells (Figure 1; Figure S4 in Supplementary Material). Integrity of the labeled antibodies was confirmed with non-reducing SDS Page (Figure S3C in Supplementary Material).

The difference in the maternal-fetal transport of infrared labeled antibody was not associated with different antibody concentrations in the blood at the time point when the transplacental transport was performed (Figure S5A in Supplementary Material). Only the DA-IgG antibody showed some trend to reduced concentration at 24 h (Figure S5B in Supplementary Material).

### Effect of Mutation on FcRn Binding

Because the function of FcRn involves IgG binding at pH 6, and antibody release at pH 7.2, we assessed all antibodies for pH-dependent binding to mouse FcRn.

**Figure 5:**
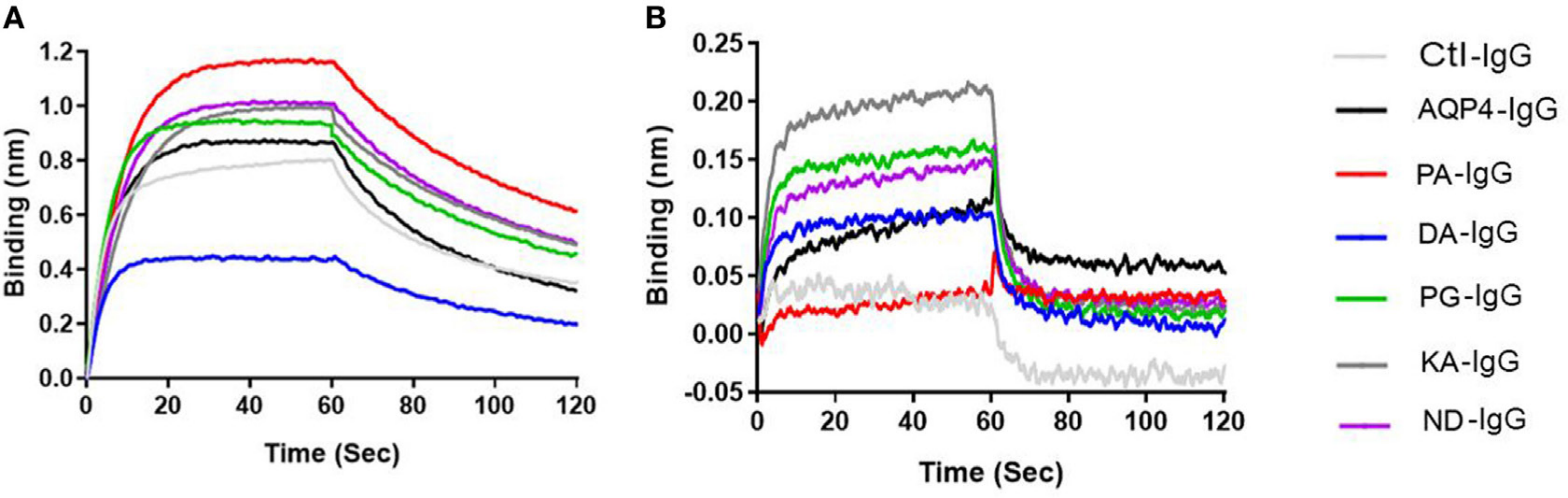
Antibodies binding to mouse FcRn. ForteBio kinetic. Ctl-IgG and AQP4–IgG and Fc-mutated antibodies (6 µg/ml) were loaded to the sensors. The sensors were exposed to 2 nM FcRn solution for association (0–60 s) and for dissociation solution (60–120 s). Sensorgrams showing the association and dissociation steps. **(A)** pH 6 and **(B)** pH 7.2. See Table 2 for Rmax and Req of all antibodies. Each antibody was analyzed three independent times and representatives are shown.

As shown in Table 2 and Figure 5A and Figure S6 in Supplementary Material all antibodies except Fc-mutated DA-IgG showed comparable binding to mouse FcRn at pH 6. Rmax and Req, but not KD values of DA-IgG indicated reduced binding to FcRn at pH 6 compared to the wild-type AQP4 antibody.

**Table 2:** Binding of antibodies to mouse FcRn.

	pH 6	pH 7.2	pH 6	pH 7.2
	Req	Req	Rmax	Rmax
Ctl-IgG	0.76	0.03	0.82	0.128
AQP4-IgG	0.8505	0.0948	0.9599	0.1043
PA-IgG	1.1556	0.0365	1.2766	0.0365
DA-IgG	0.43	0.0482	**0.45**	0.042
**PG-IgG**	0.915	0.1512	0.9796	**0.2728**
**KA-IgG**	0.9868	0.198	1.119	**0.4056**
**ND-IgG**	1.0028	0.1355	1.1174	**0.2152**

*Binding of antibodies to mouse FcRn at an IgG concentration of 6 µg/ml. Binding is shown at equilibrium response *R*_eq_ or as the maximum binding signal R_max_. Binding was tested at pH 6 and pH 7.2. All antibodies show equal binding at pH 6, except antibody DA-IgG, which has lower binding. Antibodies with enhanced binding at pH 7.2 (ND-IgG, PG-IgG, and KA-IgG) are shown in bold. Experiments were performed three times for each antibody at pH 6 and pH 7.2 and representatives are shown for each antibody*.

As expected, all antibodies bound less well to mouse FcRn (2 µM) at pH 7.2 compared to pH 6 (Figure 5B). The three Fc-mutated antibodies with the most impaired transplacental transport (KA-IgG, ND-IgG, and PG-IgG) demonstrated elevated binding to FcRn at pH 7.2, compared to the wild-type AQP4–IgG, PA-IgG, and DA-IgG.

## Discussion

Maternal IgG is transferred to the fetus during pregnancy by binding to FcRn. In mice, access to the fetal brain is possible until around embryonic day E16.5 (16). Studies have indicated the possibility that AQP4 antibodies present within the circulation of a mother with NMO might also affect the offspring (25, 31), as has been shown for some other brain-reactive antibodies (15, 32). AQP4 antibodies act primarily through CDC to induce pathology in NMOSD patients (23).

In our study, we evaluated the transplacental transport of five Fc-mutated AQP4–IgG antibodies which lack complement activation using a well-established method as previously described (16, 17, 32).

Interestingly, we observed that four out of five antibodies containing point mutations in the Fc portion that diminish CDC effector function had impaired penetration to the fetus when injected into a pregnant dam at embryonic day E14.5.

It is known that FcRn binding is required for transport of IgG across the placenta in mouse and human. Altering a single amino acid in the Fc region of IgG can affect placental transport of IgG, through affecting the binding to FcRn (33). Crystal structure analysis as well as site-directed mutagenesis have provided some information regarding the FcRn/IgG interaction sites. Particularly, amino acid residues at position 252–256 in the C_H_2 domain and positions 310, 433, 434, and 435 in the C_H_3 domain are at or in close proximity to the FcRn binding site (34–36), therefore, altering those amino acids might result in altered FcRn binding. Indeed, it has been shown that the histidine residues H310 and H435 are critical for the pH-dependent binding of IgG to FcRn (34–36). Mutations at these residues lead to decreased binding at pH 6.

In our study the substitution DA led to, although significant, only a modest reduction of transplacental transport, which could be attributed to its reduced ability to bind FcRn at pH 6, as indicated by reduced Rmax and Req values, and a trend to reduced concentration in the blood 24 h after injection.

The Fc-mutated ND-IgG, KA-IgG, and PG-IgG had substantially diminished transplacental transport compared to AQP4–IgG1. In contrast to DA-IgG, these antibodies showed intact FcRn binding at pH 6; however, all exhibited slightly enhanced FcRn binding at pH 7.2, likely resulting in a failure to be as efficiently released as wild-type AQP4-IgG from FcRn engagement at the fetal site of the placenta, which could provide one possible explanation for limited transport.

While previous studies have shown that the mutations KA and DA and P331A (but not P331G) do not affect human FcRn binding at pH 6, and PA and N297A (but not N297D) have slightly reduced affinity at pH 6 (37), we are not aware of literature testing human IgG1 at pH 6 and pH 7.2 with those mutations for binding to mouse FcRn. Asparagine 297 is a glycosylation site (38), however, there are controversial data regarding the effect of altered glycosylation on the transplacental transport (39, 40). Moreover, a previous study has shown that human IgG1 has a different affinity for mouse compared to human FcRn (41).

Little information is available to explain the lack of transplacental transport of antibodies with intact binding to FcRn. A mutated antibody with a IgG3ΔHinge mutation failed to cross the placenta in a placenta perfusion model but this was not related to a lack of human FcRn binding (33).

It also appears that other Fc receptors might be involved in transplacental transport of IgG. Evidence for a contribution of additional receptors comes from human studies. In humans, FcγRIIb is expressed by placental villous endothelial cells, and is believed to play a role in transporting IgG across the villous endothelium. In mice, FcγRIIb has been shown to mediate IgG endocytosis and transcytosis *in vitro*, and is expressed in the placenta yolk sac vasculature, and thus could function as an additional IgG transporter (42). However, it is not clear whether this receptor is critical to transport IgG across the placenta, since fetuses of mice lacking FcγRIIb show comparable levels of IgG as wild-type mice (43).

Transport of IgG from mother to fetus can also be used for therapeutic ends by fusing the therapeutic protein to an Fc fragment. Grubb and colleagues administered β-glucuronidase (GUS) fused to an Fc region to pregnant mice in order to reverse GUS deficiency in a mouse model of mucopolysaccharidosis (44). It is also possible to envision the use of IgG with an altered Fc region to generate a therapeutic agent that will not be transported to the fetus and, therefore, will not endanger the developing fetus.

We would like to hypothesize that fusing antigen to a mutated Fc region that does not cross the placenta but has a reasonable half-life might serve as a novel therapeutic strategy in pregnant mothers, to prevent the transport of pathogenic IgG to the fetus. Fc can improve solubility, stability, and half-life of the fused molecule, and as in this study, can be manipulated to have reduced CDC or other effector mechanisms. Therefore, administrating a fusion protein consisting of antigen and mutated Fc during pregnancy to absorb pathogenic antibody could theoretically remove pathogenic antibody within the mother without affecting the developing fetus. Since immune complexes are not transported to the fetus, we speculate that the Fc fusion protein will be removed by the reticuloendothelial system in the mother or the placenta. This strategy is currently being studied. This therapeutic strategy could be used to supply decoy antigen not only for AQP4–IgG but also for other antibody mediated diseases, where the presence of antigen-specific antibodies during pregnancy can adversely affect the developing fetus.

## Ethics Statement

All animal experiments were performed in accordance with the National Institutes of Health Guidelines under protocols approved by the Institutional Animal Care and Use Committee (IACUC) and the Institutional Biosafety Committee (IBC) of the Feinstein Institute for Medical Research, Northwell Health, Manhasset, NY, USA.

## Author Contributions

SM and LB designed the study, analyzed the data, and performed the experiments and wrote the manuscript. JB and JS contributed the antibodies as well as the mutated antibodies, performed the complement assays, and participated in writing the manuscript. BD designed and oversee the study and contributed to writing the manuscript.

## Conflict of Interest Statement

The authors declare that the research was conducted in the absence of any commercial or financial relationships that could be construed as a potential conflict of interest.

## References

[1] Garty BZ Ludomirsky A Danon YL Peter JB Douglas SD. Placental transfer of immunoglobulin G subclasses. Clin Diagn Lab Immunol (1994) 1(6):667–9. 8556518 10.1128/cdli.1.6.667-669.1994 PMC368387

[2] Malek A Sager R Kuhn P Nicolaides KH Schneider H. Evolution of maternofetal transport of immunoglobulins during human pregnancy. Am J Reprod Immunol (1996) 36(5):248–55. 10.1111/j.1600-0897.1996.tb00172.x 8955500

[3] Rodewald R. pH-dependent binding of immunoglobulins to intestinal cells of the neonatal rat. J Cell Biol (1976) 71(2):666–9. 10.1083/jcb.71.2.666 11223 PMC2109747

[4] Raghavan M Bonagura VR Morrison SL Bjorkman PJ. Analysis of the pH dependence of the neonatal Fc receptor/immunoglobulin G interaction using antibody and receptor variants. Biochemistry (1995) 34(45):14649–57. 10.1021/bi00045a005 7578107

[5] Vaughn DE Bjorkman PJ. Structural basis of pH-dependent antibody binding by the neonatal Fc receptor. Structure (1998) 6(1):63–73. 10.1016/S0969-2126(98)00008-2 9493268

[6] Lencer WI Blumberg RS. A passionate kiss, then run: exocytosis and recycling of IgG by FcRn. Trends Cell Biol (2005) 15(1):5–9. 10.1016/j.tcb.2004.11.004 15653072

[7] Ober RJ Martinez C Vaccaro C Zhou J Ward ES. Visualizing the site and dynamics of IgG salvage by the MHC class I-related receptor, FcRn. J Immunol (2004) 172(4):2021–9. 10.4049/jimmunol.172.4.2021 14764666

[8] Mi W Wanjie S Lo ST Gan Z Pickl-Herk B Ober RJ Targeting the neonatal fc receptor for antigen delivery using engineered fc fragments. J Immunol (2008) 181(11):7550–61. 10.4049/jimmunol.181.11.7550 19017944 PMC2738423

[9] He W Ladinsky MS Huey-Tubman KE Jensen GJ McIntosh JR Bjorkman PJ. FcRn-mediated antibody transport across epithelial cells revealed by electron tomography. Nature (2008) 455(7212):542–6. 10.1038/nature07255 18818657 PMC2773227

[10] Vernet-der Garabedian B Lacokova M Eymard B Morel E Faltin M Zajac J Association of neonatal myasthenia gravis with antibodies against the fetal acetylcholine receptor. J Clin Invest (1994) 94(2):555–9. 10.1172/JCI117369 8040310 PMC296130

[11] Vincent A Newland C Brueton L Beeson D Riemersma S Huson SM Arthrogryposis multiplex congenita with maternal autoantibodies specific for a fetal antigen. Lancet (1995) 346(8966):24–5. 10.1016/S0140-6736(95)92652-6 7603140

[12] Oskoui M Jacobson L Chung WK Haddad J Vincent A Kaufmann P Fetal acetylcholine receptor inactivation syndrome and maternal myasthenia gravis. Neurology (2008) 71(24):2010–2. 10.1212/01.wnl.0000336929.38733.7a 19064884 PMC2676977

[13] Hon KL Leung AK. Neonatal lupus erythematosus. Autoimmune Dis (2012) 2012:301274. 10.1155/2012/301274 22973504 PMC3437607

[14] Brimberg L Sadiq A Gregersen PK Diamond B. Brain-reactive IgG correlates with autoimmunity in mothers of a child with an autism spectrum disorder. Mol Psychiatry (2013) 18(11):1171–7. 10.1038/mp.2013.101 23958959

[15] Brimberg L Mader S Jeganathan V Berlin R Coleman TR Gregersen PK Caspr2-reactive antibody cloned from a mother of an ASD child mediates an ASD-like phenotype in mice. Mol Psychiatry (2016) 21(12):1663–71. 10.1038/mp.2016.165 27698429 PMC5583730

[16] Braniste V Al-Asmakh M Kowal C Anuar F Abbaspour A Toth M The gut microbiota influences blood-brain barrier permeability in mice. Sci Transl Med (2014) 6(263):263ra158. 10.1126/scitranslmed.3009759 25411471 PMC4396848

[17] Lee JY Huerta PT Zhang J Kowal C Bertini E Volpe BT Neurotoxic autoantibodies mediate congenital cortical impairment of offspring in maternal lupus. Nat Med (2009) 15(1):91–6. 10.1038/nm.1892 19079257 PMC2615794

[18] Singer HS Morris C Gause C Pollard M Zimmerman AW Pletnikov M. Prenatal exposure to antibodies from mothers of children with autism produces neurobehavioral alterations: a pregnant dam mouse model. J Neuroimmunol (2009) 211(1–2):39–48. 10.1016/j.jneuroim.2009.03.011 19362378

[19] Braunschweig D Golub MS Koenig CM Qi L Pessah IN Van de Water J Maternal autism-associated IgG antibodies delay development and produce anxiety in a mouse gestational transfer model. J Neuroimmunol (2012) 252(1–2):56–65. 10.1016/j.jneuroim.2012.08.002 22951357 PMC4096980

[20] Tan CT Mao Z Qiu W Hu X Wingerchuk DM Weinshenker BG International consensus diagnostic criteria for neuromyelitis optica spectrum disorders. Neurology (2016) 86(5):491–2. 10.1212/WNL.0000000000002366 26833940

[21] Bradl M Lassmann H Oligodendrocytes: biology and pathology. Acta Neuropathol (2010) 119(1):37–53. 10.1007/s00401-009-0601-5 19847447 PMC2799635

[22] Bennett JL Lam C Kalluri SR Saikali P Bautista K Dupree C Intrathecal pathogenic anti-aquaporin-4 antibodies in early neuromyelitis optica. Ann Neurol (2009) 66(5):617–29. 10.1002/ana.21802 19938104 PMC3180961

[23] Papadopoulos MC Bennett JL Verkman AS. Treatment of neuromyelitis optica: state-of-the-art and emerging therapies. Nat Rev Neurol (2014) 10(9):493–506. 10.1038/nrneurol.2014.141 25112508 PMC4229040

[24] Ratelade J Asavapanumas N Ritchie AM Wemlinger S Bennett JL Verkman AS. Involvement of antibody-dependent cell-mediated cytotoxicity in inflammatory demyelination in a mouse model of neuromyelitis optica. Acta Neuropathol (2013) 126(5):699–709. 10.1007/s00401-013-1172-z 23995423 PMC3890328

[25] Nour MM Nakashima I Coutinho E Woodhall M Sousa F Revis J Pregnancy outcomes in aquaporin-4-positive neuromyelitis optica spectrum disorder. Neurology (2016) 86(1):79–87. 10.1212/WNL.0000000000002208 26581304 PMC4731292

[26] Palmer MH Hoffmann SV Jones NC Coreno M de Simone M Grazioli C A combined theoretical and experimental study of the valence and Rydberg states of iodopentafluorobenzene. J Chem Phys (2017) 146(17):174301. 10.1063/1.4981919 28477584

[27] Tradtrantip L Zhang H Saadoun S Phuan PW Lam C Papadopoulos MC Anti-aquaporin-4 monoclonal antibody blocker therapy for neuromyelitis optica. Ann Neurol (2012) 71(3):314–22. 10.1002/ana.22657 22271321 PMC3314396

[28] Idusogie EE Presta LG Gazzano-Santoro H Totpal K Wong PY Ultsch M Mapping of the C1q binding site on rituxan, a chimeric antibody with a human IgG1 Fc. J Immunol (2000) 164(8):4178–84. 10.4049/jimmunol.164.8.4178 10754313

[29] Sazinsky SL Ott RG Silver NW Tidor B Ravetch JV Wittrup KD. Aglycosylated immunoglobulin G1 variants productively engage activating Fc receptors. Proc Natl Acad Sci U S A (2008) 105(51):20167–72. 10.1073/pnas.0809257105 19074274 PMC2629253

[30] Mader S Lutterotti A Di Pauli F Kuenz B Schanda K Aboul-Enein F Patterns of antibody binding to aquaporin-4 isoforms in neuromyelitis optica. PLoS One (2010) 5(5):e10455. 10.1371/journal.pone.0010455 20463974 PMC2864757

[31] Saadoun S Waters P Leite MI Bennett JL Vincent A Papadopoulos MC. Neuromyelitis optica IgG causes placental inflammation and fetal death. J Immunol (2013) 191(6):2999–3005. 10.4049/jimmunol.1301483 23935196 PMC4161708

[32] Wang L Zhou D Lee J Niu H Faust TW Frattini S Female mouse fetal loss mediated by maternal autoantibody. J Exp Med (2012) 209(6):1083–9. 10.1084/jem.20111986 22565825 PMC3371726

[33] Mathiesen L Nielsen LK Andersen JT Grevys A Sandlie I Michaelsen TE Maternofetal transplacental transport of recombinant IgG antibodies lacking effector functions. Blood (2013) 122(7):1174–81. 10.1182/blood-2012-12-473843 23843496

[34] Grevys A Bern M Foss S Bratlie DB Moen A Gunnarsen KS Fc engineering of human IgG1 for altered binding to the neonatal Fc receptor affects Fc effector functions. J Immunol (2015) 194(11):5497–508. 10.4049/jimmunol.1401218 25904551 PMC4432726

[35] Kim JK Firan M Radu CG Kim CH Ghetie V Ward ES. Mapping the site on human IgG for binding of the MHC class I-related receptor, FcRn. Eur J Immunol (1999) 29(9):2819–25. 10.1002/(SICI)1521-4141(199909)29:09<2819::AID-IMMU2819>3.0.CO;2-6 10508256

[36] Burmeister WP Huber AH Bjorkman PJ. Crystal structure of the complex of rat neonatal Fc receptor with Fc. Nature (1994) 372(6504):379–83. 10.1038/372336a0 7969498

[37] Shields RL Namenuk AK Hong K Meng YG Rae J Briggs J High resolution mapping of the binding site on human IgG1 for Fc gamma RI, Fc gamma RII, Fc gamma RIII, and FcRn and design of IgG1 variants with improved binding to the Fc gamma R. J Biol Chem (2001) 276(9):6591–604. 10.1074/jbc.M009483200 11096108

[38] Mimura Y Sondermann P Ghirlando R Lund J Young SP Goodall M Role of oligosaccharide residues of IgG1-Fc in Fc gamma RIIb binding. J Biol Chem (2001) 276(49):45539–47. 10.1074/jbc.M107478200 11567028

[39] Dashivets T Thomann M Rueger P Knaupp A Buchner J Schlothauer T. Multi-angle effector function analysis of human monoclonal IgG glycovariants. PLoS One (2015) 10(12):e0143520. 10.1371/journal.pone.0143520 26657484 PMC4676693

[40] Wilcox CR Holder B Jones CE. Factors affecting the FcRn-mediated transplacental transfer of antibodies and implications for vaccination in pregnancy. Front Immunol (2017) 8:1294. 10.3389/fimmu.2017.01294 29163461 PMC5671757

[41] Abdiche YN Yeung YA Chaparro-Riggers J Barman I Strop P Chin SM The neonatal Fc receptor (FcRn) binds independently to both sites of the IgG homodimer with identical affinity. MAbs (2015) 7(2):331–43. 10.1080/19420862.2015.1008353 25658443 PMC4622529

[42] Kim J Mohanty S Ganesan LP Hua K Jarjoura D Hayton WL FcRn in the yolk sac endoderm of mouse is required for IgG transport to fetus. J Immunol (2009) 182(5):2583–9. 10.4049/jimmunol.0803247 19234152 PMC2676880

[43] Mohanty S Kim J Ganesan LP Phillips GS Hua K Jarjoura D IgG is transported across the mouse yolk sac independently of FcgammaRIIb. J Reprod Immunol (2010) 84(2):133–44. 10.1016/j.jri.2009.10.008 20015554 PMC3050502

[44] Grubb JH Vogler C Tan Y Shah GN MacRae AF Sly WS. Infused Fc-tagged beta-glucuronidase crosses the placenta and produces clearance of storage in utero in mucopolysaccharidosis VII mice. Proc Natl Acad Sci U S A (2008) 105(24):8375–80. 10.1073/pnas.0803715105 18544647 PMC2448844

