# Supplementary material for: Mutations of Recombinant Aquaporin-4 Antibody in the Fc Domain Can Impair Complement-Dependent Cellular Cytotoxicity and Transplacental Transport
Source: Front Immunol. 2018 Jul 13;9:1599. doi: 10.3389/fimmu.2018.01599 (PMC6053506; doi:10.3389/fimmu.2018.01599)
Supplement: Supplementary file 1 [file data_sheet_1.docx]

Supplementary Material

**Mutations of recombinant aquaporin-4 antibody in the Fc domain can impair complement dependent cellular cytotoxicity and transplacental transport**

Simone Mader^1&^, Lior Brimberg^1&^, John N. Soltys^2^, Jeffrey L. Bennett^3^_,_ Betty Diamond^1*^

^1^The Feinstein Institute for Medical Research, The Center for Autoimmune, Musculoskeletal and Hematopoietic Diseases, Northwell Health System, Manhasset, NY, United States

^2^Medical Scientist Training and Neuroscience Graduate Training Programs, University of Colorado Denver School of Medicine, Aurora, CO 80045, United States

^3^Departments of Neurology and Ophthalmology, Program in Neuroscience, University of Colorado Denver School of Medicine, Aurora, CO 80045, United States

*** Correspondence:**Betty Diamond
[bdiamond@northwell.edu](mailto:bdiamond@northwell.edu)

**^&^These authors contributed equally**

## Supplementary Figures


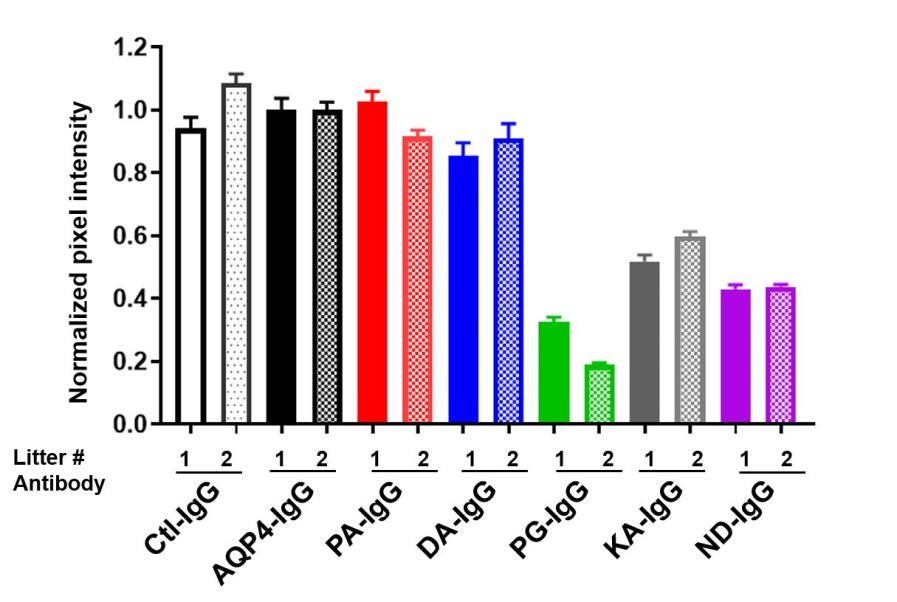


**Supplementary Figure 1: Quantification of infrared signal of transplacental transport**

Normalized pixel intensity is presented by litter. Values of Data of each embryo normalized to the average pixel intensity of an AQP4-IgG exposed litter.  Replicates confirmed our initial observation, with PG-IgG, KA-IgG and ND-IgG values showing reduced transplacental transport.

**
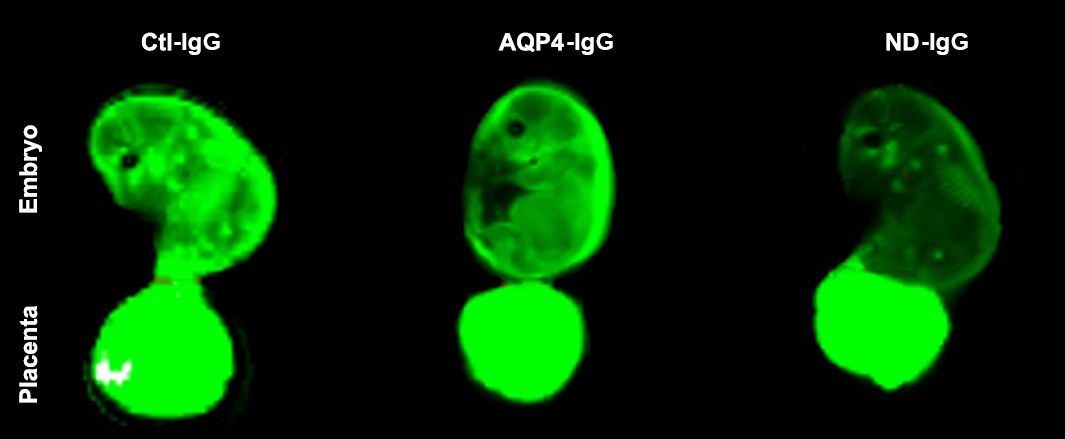
**

**Supplementary Figure 2:** Similar levels of infrared signal in the placenta of the Fc mutated antibody ND-IgG, compared to AQP4-IgG and Ctl-IgG, despite limited transplacental transport to the fetus.

**
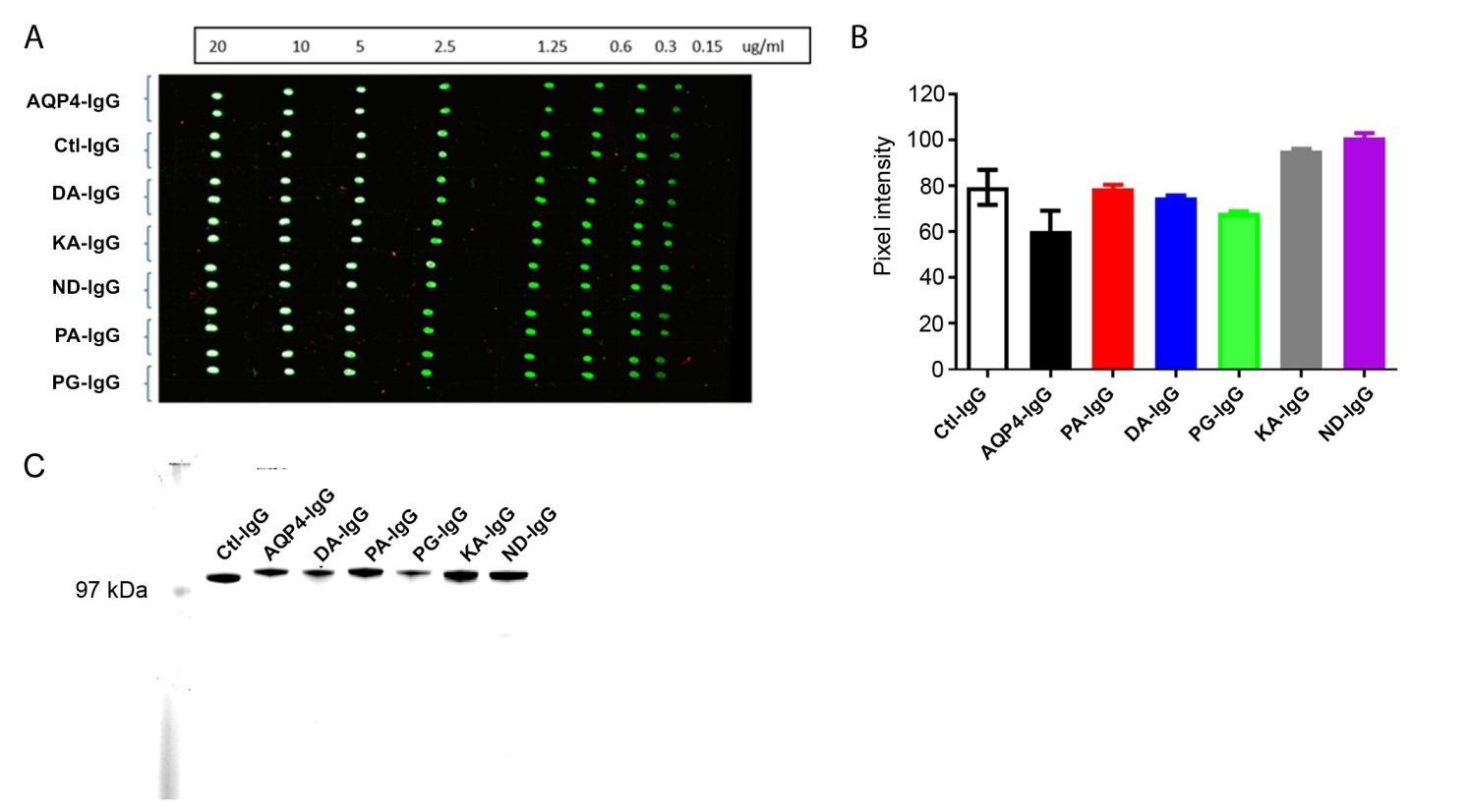
**

**Supplementary Figure 3: Confirmation of equal labelling of recombinant antibodies and antibody integrity.**

Comparable level of infrared signal intensity of all the antibodies used for the in vivo experiment is seen by dot blot, using serial dilutions of each antibody starting from 20 μg/ml (Figure 1A). Quantification of the infrared signal intensity was performed in duplicates and is shown for an antibody concentration of 1.25 μg/ml (B). Antibodies were analyzed for integrity after labelling using a non-reducing SDS Page (C).

**
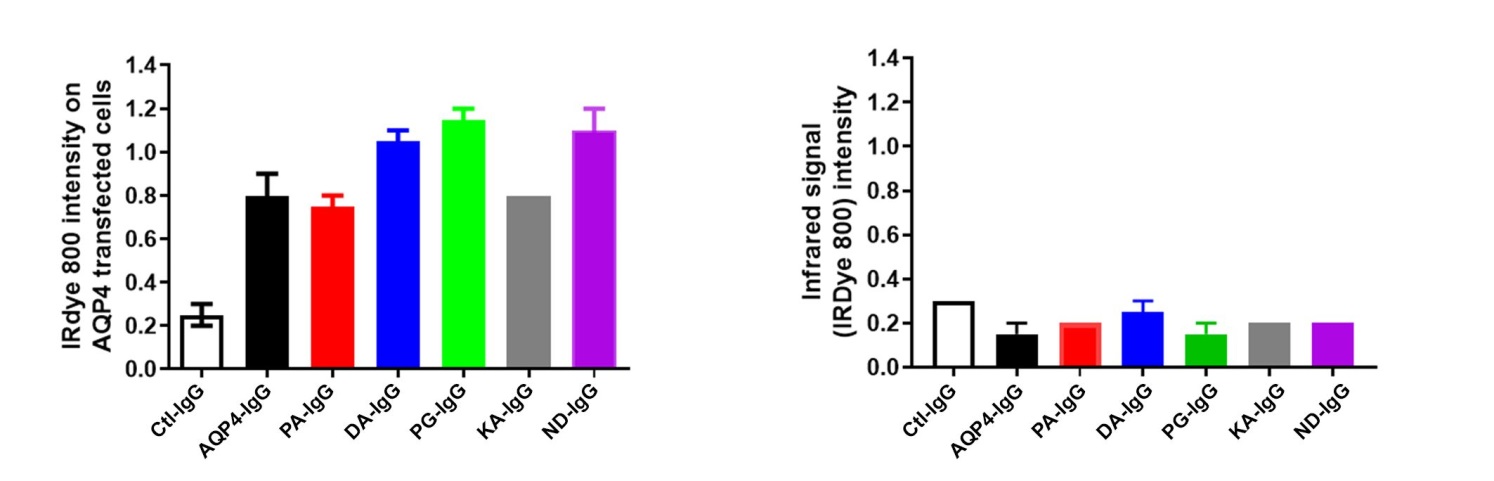
**

**Supplementary Figure 4: Binding of infrared labelled AQP4-IgG and mutants to AQP4 transfected cells (in situ)**

AQP4-IgG and all the mutated antibodies bind to AQP4 transfected HEK cells using a live cell based assay. The isotype control antibody Ctl-IgG does not bind to AQP4 transfected cells (left graph). Antibodies show reduced and equivalent binding to un-transfected cells (Figure 3B, right graph).

**
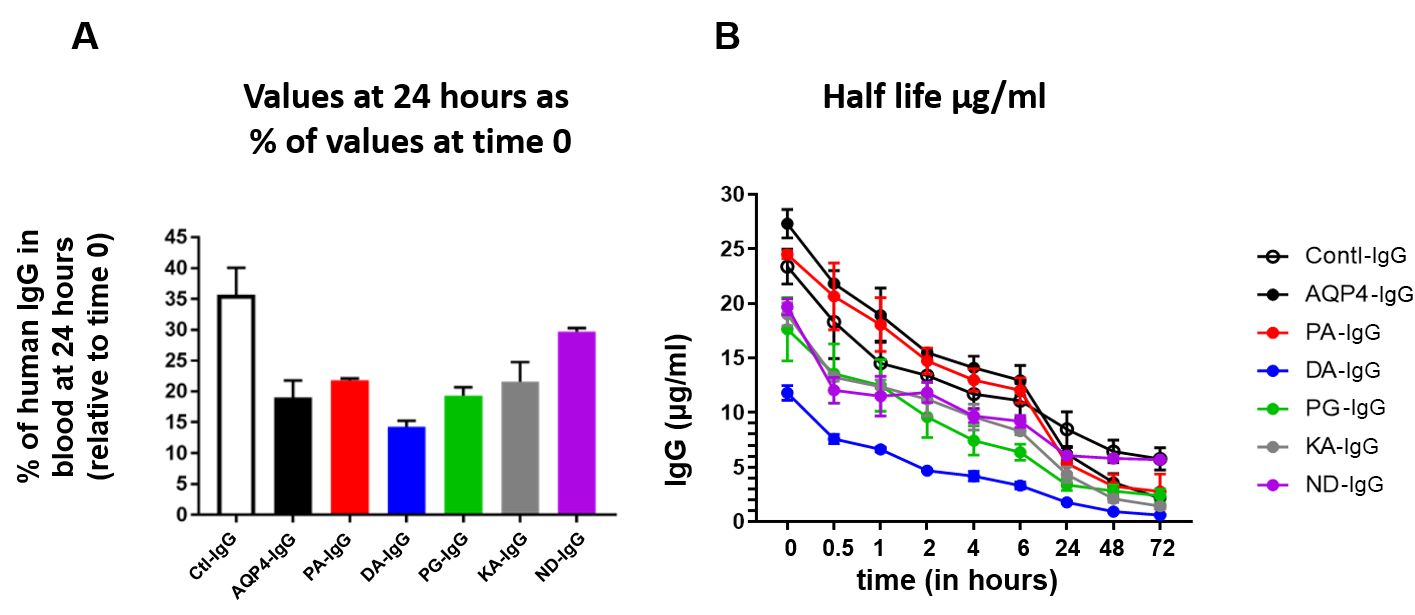
**

**Supplementary Figure 5: Serum half life of antibodies.**

Following injection with Ctl-IgG, AQP4-IgG and mutated IgG, we measured human IgG concentrations at (A) 24 hours, the time point the transplacental transport was analyzed. Human IgG in the serum of mice at 24 hours is presented as a percentage of concentration at time 0 (3 min after injection). (B) The observed mean serum antibody concentration (μg/ml) and the standard error for each antibody (n=3) were plotted as a function of hours.


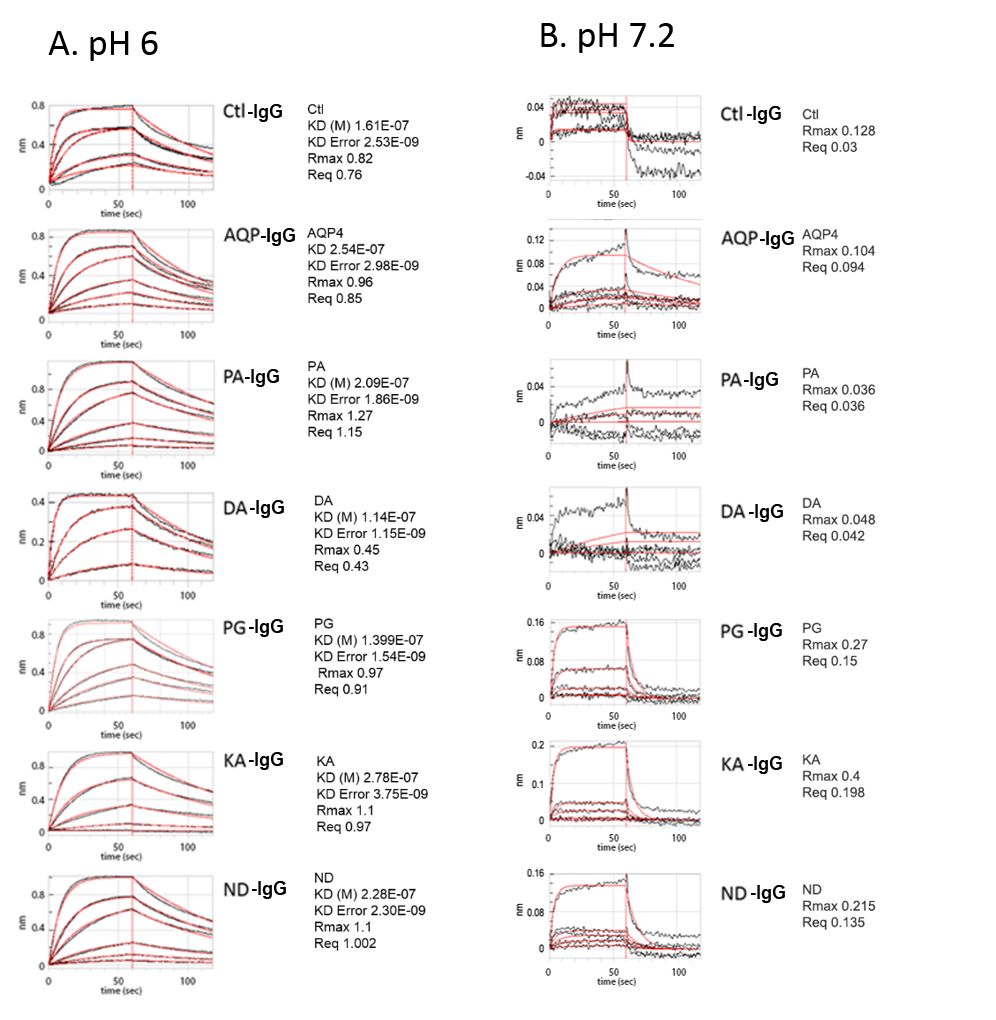


**Supplementary Figure 6: Kinetic analysis at pH6 (A) or pH7.2 (B) of Control, AQP4 and mutated antibodies binding to FcRn.** FcRn concentration was added as a 2 fold dilution starting at 2 μM. Each overlay plot shows representative example of the measured data (black lines) and the global fit (red lines) of a representative experiment. The experiment was repeated independently three times. KD values and KD Error are not presented for the Kinetic analysis at pH7.2 (B) given the low readings.
